# Ruderal *Tithonia diversifolia* inclusion in sheep diets: impacts on digestibility and greenhouse gas emissions

**DOI:** 10.1007/s11250-025-04664-5

**Published:** 2025-09-26

**Authors:** Simón Pérez-Márquez, Vagner Ovani, Ângela Maria Quintão Lana, Helder Louvandini, Adibe Luiz Abdalla, Rogério Martins Maurício

**Affiliations:** 1 Departamento de Produção Animal, Universidade Federal de Minas Gerais, Belo Horizonte, MG Brazil; 2 Rothamsted Research, North Wyke, Okehampton, Devon, EX20 2SB UK; 3 Laboratório de Nutrição Animal, Centro de Energia Nuclear na Agricultura, Universidade de São Paulo, Piracicaba, SP CEP: 13400-970 Brazil; 4 Departamento de Biotecnologia, Universidade Federal de São João del Rei, São João del Rei, MG Brazil

**Keywords:** Methane, Nitrous oxide, Carbon dioxide, Mexican sunflower, Sustainability, Santa ines

## Abstract

Emissions from ruminant livestock represent an important component of agricultural greenhouse gas output. The sector, however, has substantial potential for emission reduction through improved practices. *Tithonia diversifolia* (TD), a shrub that thrives in low-fertility soils, offers promise as a sustainable feed alternative. This study explores whether ruderal TD, accessible but with variable nutritional quality, can be used to reduce enteric methane (CH_4_) emissions and nitrogen (N) excretion in sheep, offering a low-input strategy for enhancing ruminant sustainability. Eight adult rams were used to evaluate diets with 4 increasing levels of TD hay on carbon dioxide (CO_2_), CH_4_, nitrous oxide (N_2_O) and ammonia (NH_3_) emissions, apparent digestibility, and fermentation parameters. The animals received four increasing levels of TD hay (0, 90, 270, 450 g kg^− 1^ DM) in a diet based on Tifton 85 hay, soybean meal, and ground corn. Feeding sheep with ruderal TD had no effects on intake and N balance but reduced digestibility of dry matter, organic matter, neutral and acid detergent fiber, while crude protein digestibility remained unaffected. There was also a decrease in acetate and ruminal N-NH_3_ concentrations, alongside an increase in iso-acid proportions. CO_2_, CH_4_, N_2_O and NH_3_ emissions were consistent across diets, averaging 98.05 gCO_2_ kg^− 1^ DMI, 9.3 gCH_4_ kg^− 1^ DMI, 2.62 gN_2_O kg^− 1^ excreted N, and 37.8 gNH_3_ kg^− 1^ excreted N. In conclusion, incorporating ruderal TD into sheep diets reduced nutrient digestibility and ruminal fermentation but did not impact feed intake, protein digestibility, or greenhouse gas emissions.

## Introduction

According to the latest international panel for climate change report, both methane (CH_4_) and nitrous oxide (N_2_O) emissions from the agriculture sector continue to increase, with enteric fermentation, manure application, nitrogen (N) deposition and fertilizer use, being the main driver (Nabuurs et al. 2022) s. Nonetheless, in this same report, is also mentioned that the agriculture, forestry and other land use sector can offer up to 30% of the global mitigation potential needed to maintain the earth temperature below 2 °C above pre-industrial levels. Various studies indicate substantial differences in emission intensity across comparable ruminant production systems, highlighting the sector’s potential for enhancement (Gerber et al. 2013; Poore and Nemecek 2018). It is proposed that adopting the methods of the top 10% of producers could significantly lower greenhouse gas (GHG) emissions from livestock without affecting output (Bajželj et al. 2014). Improved management of grasslands and livestock, agroforestry and sustainable intensification are measures that offer significant near-term mitigation potential (Nabuurs et al. 2022).

In livestock management and sustainable intensification, utilizing feed alternatives that do not compete with human food resources is crucial (Mottet et al. 2017). Ruminants can convert fibrous plant materials into high-quality protein, yet a significant amount of human-edible grain is still used in ruminant diets (Mottet et al. 2017). Introducing alternative forage plants, particularly locally available shrubs, is a promising strategy to reduce CH₄ emissions (Palangi et al. 2022) while minimizing reliance on external inputs and supporting low-input production systems (Herrero et al. 2016; Distel et al. 2020).

*Tithonia diversifolia* (Hemsl.) A. Gray (TD), a shrub plant belonging to the *Asteracea* family, holds great potential as a feed source for ruminants. It is already being routinely used in countries such as Colombia, Cuba, and Mexico (Mahecha et al. 2008; Rivera et al. 2016; Ribeiro et al. 2016). Tithonia is a forage plant with a global distribution that thrives in low-fertility soils and has been reported to possess characteristics such as nutrient accumulation, improved soil phosphorus availability, soil extractable aluminium reduction, and acidic soil tolerance (Olivares et al. 2002; Cong and Merckx 2005; Adesodun et al. 2010; Ojeniyi et al. 2012; Ovani et al. 2024).

*Tithonia diversifolia*’s potential to impact greenhouse gas emissions from livestock activities have also been investigated. It has been mentioned that its presence in ruminant diets could help mitigate ruminal methane emissions (Rivera et al. 2022; Krüger et al. 2024), and although research specifically pertaining the effects of TD on N_2_O emissions is limited in the existing literature (Rivera et al. 2023); works have investigated the N metabolism of ruminants fed with TD (Ramírez-Rivera et al. 2010; Durango et al. 2021; Cardona et al. 2022) and reported that that its presence in the ruminant diet has the potential to enhance N retention. As N presence in the excreta is directly correlated with N_2_O emissions (Rivera and Chará 2021), alterations in dietary protein digestibility through the inclusion of TD may influence the emission intensity of ruminants.

*Tithonia diversifolia* is also commonly found in various environments through natural regeneration or accidental introduction, including urban areas (Gavilanes and D’Angieri Filho 1991; Val Diaz et al. 2017; Balangcod and Balangcod 2020; Durán-Puga et al. 2020). This widespread availability presents an opportunity to utilize TD as a smart supplement (Eisler et al. 2014; Jia et al. 2019) or a low-input, low-maintenance feeding alternative in ruminant diets. However, the nutritional quality of TD is highly variable and heavily dependent on its phenological stage (Ajao and Moteetee 2017; Uu-Espens et al. 2023; Ovani et al. 2025), with the best results obtained when the plant is harvested before flowering (Calsavara et al. 2016; Ruíz et al. 2024), which may not always be the case when using wild-grown or ruderal material.

Given the plant’s ease of growth, it is worth investigating whether TD plants growing in ruderal areas retain their beneficial characteristics and can be effectively used in animal production. Therefore, the objective of this study was to evaluate the effects of feeding sheep with ruderal TD, on their digestibility and GHG emissions. Specifically, the study aimed to determine whether feeding sheep with ruderal TD could be a viable strategy to reduce enteric CH_4_, and N excretion.

## Materials and methods

### Location

The TD was collected from different areas around the city of São João del Rei in the state of Minas Gerais, Brazil (21°08′09″S 44°15′36″W, average annual ground temperature of 19.3 °C, and relative humidity of 73%), during the period of June to November 2017. The animal experiment and the laboratory analyses were all conducted at the Animal Nutrition Laboratory of the Nuclear Energy in Agriculture Center (CENA) at the University of São Paulo in Piracicaba, São Paulo, Brazil.

### Experimental diets

The diets used in this study have also been described by Pérez-Márquez et al. (2023) in an in vitro approach. A control diet consisting of 400 g kg^− 1^ soybean meal and corn grain, and 600 g kg^− 1^ Tifton 85 hay (*Cynodon spp*) (TD0), was compared against three increasing levels of TD hay (90, 270, and 450 g kg^− 1^ on a dry matter [DM] basis) as a replacement for Tifton hay (TD9, TD27 and TD45, respectively). The diets were formulated to meet the maintenance requirements for sheep (National Research Council 2007) with a 25 g kg^− 1^ DM intake of liveweight per day and were balanced to be iso-proteic and iso-fibrous using the values for DM, crude protein (CP), neutral detergent fiber (NDF) of the components. The ingredients and chemical composition of the diets are described in Tables 1 and 2.

**Table 1:** Chemical composition of diet’s ingredients (g kg− 1)

	Soybean Meal	Maize	Tifton 85 hay	*T. diversifolia* hay
Dry matter	897.89	887.00	903.04	887.07
Neutral detergent fiber	150.00	111.48	735.81	677.71
Acid detergent fiber	110.96	34.20	360.23	566.62
Crude protein	479.23	127.14	140.45	78.84
Ash	69.19	12.79	98.96	111.35

**Table 2:** Ingredient proportion (g kg^− 1^) and chemical composition (g kg^− 1^ DM) of diets containing increasing levels of *Tithonia diversifolia*

Ingredient (g kg^− 1^)	TD0	TD9	TD27	TD45	
*Tithonia diversifolia*	0	90	270	450	
Tifton 85	600	510	330	150	
Maize	263	254.7	246.2	234.9	
Soybean meal	137.0	142.6	153.8	165.1	
Chemical Component (g kg^− 1^ DM)					
Dry matter (g kg^− 1^)	910.6	906.9	906.4	905.7	
Neutral detergent fiber ^ab^	409.0	396.6	393.3	392.7	
Non-structural carbohydrates	342.7	366.1	367.0	353.8	
Acid detergent fiber ^b^	299.2	300.3	312.7	345.8	
Crude protein	163.0	158.6	156.5	156.3	
Lignin ^b^	75.6	83.92	98.48	129.0	
Ash	61.5	71.9	78.6	89.2	
Ether extract	23.7	18.0	17.67	17.5	
Crude energy (kcal gDM^− 1^)	3748	3713	3715	3697	

^a^assayed using thermostable amylase and ^b^ expressed as exclusive of residual ash

*Tithonia diversifolia* at flowered stage (~ 100 days old) was collected from different areas around the city of São João del Rei in the state of Minas Gerais, Brazil. The entire plant (leaves and stems) was harvested at about 80 cm from the base, chopped, and then sun-dried until it was dry to the touch. The dried TD was then transported to the CENA’s animal nutrition laboratory where it was further ground to pass through a 1 cm sieve to facilitate mixing with the other components of the diet. Tifton-85 hay was initially chopped into 3 cm particles and then further ground to 1 cm. Corn grain was also broken down in a grinder without the use of a sieve. Soybean meal was used without prior mechanical grinding. The ingredients were mixed using a 500 kg capacity mixer for 15 min to form the complete diets, which were then individually stored in 200-liter plastic drums in a dry and sun-free location.

After mixing the ingredients for each diet, a sample of each one was ground to 1 mm (using a Willey mill) for determination of chemical composition. The guidelines of AOAC (2011) were followed for determination of DM content (method 934.01), CP (method 2001.11), EE (method 2003.5), and ash fraction (method 942.05). Neutral detergent fiber (evaluated using thermostable amylase and sulphite and expressed as ash-free residual), ADF (expressed as ash-free residual), and lignin were evaluated according to Van Soest et al. (1991) adapted by Mertens et al. (2002). The Non-structural carbohydrates (NSC) contents were calculated by the equation: NSC = 100 - (CP + NDF + EE + ash) (Sniffen et al. 1992). Neutral detergent indigestible nitrogen (NIDN) and acid detergent indigestible nitrogen (NIDA) were determined according to the methods N-004/1 and N-005/1, respectively, of the INCT-CA (Detmann et al. 2012) and expressed as a percentage of dry matter and total N.

### Animals and experimental design

Eight castrated male sheep (Santa Ines breed), adults, with an average body weight and standard deviation of 70 ± 13.4 kg, fitted with ruminal cannula from the herd of the animal nutrition laboratory of the CENA/USP, were used. The sheep were paired by bodyweight and randomly allocated to one of the four diets. Each animal underwent a 14-day adaptation to the diets before the evaluation. The animals’ responses to the diet were evaluated for four periods in a crossover design with 10-day observation periods, and 14 days washout periods. Water and mineral mixture were provided *ad libitum* during whole experimental period.

### Digestibility trial

The sheep were housed in metabolic crates (0.8 × 1.2 m) equipped with a feeder, water and mineral salt troughs, and trays for feces and urine collection. The sheep were evaluated over a period of seven days, including two days for animal adaptation to the crates and five days for sample collection. The diet was provided daily in two meals: one at 8:00 and another at 16:00, both in equal proportions. The amount of feed offered was adjusted daily to ensure 10% leftovers (fresh matter basis). Diet samples were collected from each evaluation period. The leftover and feces samples from each animal were collected daily before the first meal, weighed on an electronic scale (with a precision of 5 g), sampled (10%) (forming a pool of leftovers and a pool of feces for each animal per period), and stored in a freezer at -20 °C for subsequent bromatological analysis using the previously described analysis methodologies.

Nutrient’s apparent digestibility was determined according to the equation described by McDonald et al. (2011).

$$\:AD\:of\:X=\frac{{X}_{intake}-{X}_{excreted}}{{X}_{intake}}$$

Where: AD = Apparent digestibility (g/g); X = evaluated nutrient.

At the end of the four experimental periods, the offered, leftover, and faecal samples were thawed (at room temperature), weighed and dried in a forced-air circulation oven at 55 °C to a constant weight and then ground for determination of DM, organic matter (OM), CP, NDF, ADF, and lignin.

### Urine collection

Before providing the first daily meal, urine was collected from plastic sampling trays containing 100 mL of 10% sulfuric acid to prevent ammonia volatilization (Knowlton et al. 2010). The total volume of urine was measured every day, and a 10% aliquot was sampled to form a urine pool per animal per evaluation period. Urine samples were stored in a freezer at -20 °C for subsequent total N analysis.

### N balance

The calculation of N balance was performed after determining the total N present in the offered and excreta samples using the AOAC (2011) method 954.01 for N determination and following the equation described below:

$$\begin{aligned}\:{N}_{retained\:\left({g\:day}^{-1}\right)}&={N}_{ingested}-\left({N}_{excreted\:in\:feces\:}+\:{N}_{excreted\:in\:urine}\right)\end{aligned}$$

Where: Nretained = average amount of N retained by the animal; Ningested = average amount of N ingested by the animal; Nexcreted = average amount of N excreted in urine + average amount of N excreted in feces.

### Respiration chambers

After the digestibility trial, the eight animals were placed in individual respiration chambers adapted with a ventilation system for gas measurement. A total of 10 metal chambers (157 × 71 × 167 cm - volume 1.9 m^3^) described by Abdalla et al. (2012) were used for the experiment. The chambers were connected through pipelines to a 16-port distribution manifold (A0311, 16-Port distribution manifold, Picarro, INC., Santa Clara, CA - USA) that allowed the selection of emissions from each chamber (one at a time) and sent them in real-time to a gas concentration analyser (G2508 Picarro Inc, Santa Clara, California - USA) using cavity ring-down spectroscopy (CRDS) technology for simultaneous determination of CO_2_, CH_4_, N_2_O (ppm - µmol/mol (v/v), and NH_3_ (ppb - nmol / mol (v/v) concentrations at a frequency of approximately one hertz. Two of the respiration chambers were left empty (blanks), and an extra pipeline measured the gas concentrations of the room where the chambers were allocated for subsequent emission corrections in relation to the blank and ambient air. Thus, a total of eight chambers with sheep, two blanks, and one ambient air (eleven units) were measured. Each chamber was assigned to an individual sheep for the whole experiment, the diet treatments were randomly assigned to each sheep every period.

The gas emission evaluation was carried out over a period of three days, with one day for adaptation and two for collection. Feed was provided to the animals at 8:00 and 16:00, and the temperature, internal humidity, and air flow rate (m/s) of each chamber were measured during the day at 8, 11, 14, 17, and 20 h. During the two days of emission evaluation, measurements were autonomously taken one chamber at a time, where the gas concentrations were measured for three uninterrupted minutes in each chamber. Every period, gas measurements were taken sequentially from chamber 1 to chamber 11, in cycles of 33 min (3 min x 11 chambers), starting at 8 a.m. and ending at approximately 6 a.m. the following day (22 h approx.). Before providing the morning feed on the second day, the feces and urine in the chambers were removed, and feed leftovers were removed and weighed. After cleaning the chambers, measurements were resumed in the same manner as on the first day.

### Calculations for emission determination

The raw data obtained from the gas analyser during the four measurement periods were evaluated using R Studio software (R Core Team 2021) using R packages ‘dplyr’ (Wickham et al. 2023), ‘lubridate’ (Grolemund and Wickham 2011) and ‘tidyr’ (Wickham et al. 2024). For each chamber, the first 60 s of each 3-minute measurement period were excluded to avoid contamination from residual gases originating from the previous chamber measurement. Then, using the values of the remaining 2 min, a median of the gas was calculated to avoid the influence of any extreme values or aberrations in the data collection. This median was considered a concentration point. For each chamber, an average (per gas) of all the concentration points after 22 h of measuring was obtained. This was considered the average daily concentration.

### Daily gas emission rates

Based on the average daily concentration of emitted gas and using the data obtained from the digestibility assay for each animal and the ideal gas law at standard conditions, the emissions were expressed in grams of gas per day (g d^− 1^), grams of gas per kilogram of live weight (g kg^− 1^ LW), and grams of gas per kilogram of DM intake (g kg ^− 1^ DMI). The emissions of N_2_O and NH_3_ were also expressed as grams of gas per kilogram of excreted N (g kg ^− 1^ N excreted), using the N balance values.

### Ruminal fermentation parameters

On the final day of the gas emission quantification assay, two hours after the morning feed, a sample of ruminal fluid from the sheep was collected via rumen canula. Each sample was divided into three aliquots for the determination of short-chain fatty acids (SCFAs), ammonia N (N-NH_3_), and protozoa populations. The determination of SCFAs was conducted using gas chromatography following the methodology and equipment described by Lima et al. (2018). The concentration of N-NH_3_ was determined using the micro-Kjeldahl method, involving steam distillation with a 5% sodium tetraborate solution for the reaction and condensation of N-NH_3_, boric acid as the receiving solution, and 0.01 N sulfuric acid for titration. Protozoa populations were determined by visual examination under a light microscope using a Neubauer chamber; 2 mL of rumen fluid were fixated in 4 mL of a methyl formaline solution following the methodologies of Ogimoto and Imai (1981); Dehority (1993) and Göçmen et al. (2001).

### Statistical analysis

For most of the responses, the data were analysed as crossover design, using 4 treatments (TD inclusion level) being evaluated over 4 periods (rows) and 8 animals (columns) with a 14 -day washout period. For daily gas emission rates, the average daily emission values from each 22 h period were considered a replicate for that observation within that treatment.

Shapiro-Wilk normality test and Bartlett’s homoscedasticity test were conducted. Data were transformed, if necessary, using exponential, logistic, or square root transformations. Pearson correlation tests and regression analysis were performed to evaluate the effect of the increasing level of TD in the assessed variables. The treatment means were also compared using orthogonal contrasts, comparing the TD inclusion treatments against the TD-free diet; TD0 vs. TD9 (1,-1,0,0), TD0 vs. TD27 (1,0,-1,0), TD0 vs. TD45 (1,0,0,-1). Results were considered significant when the p value was inferior to 0.05.

The statistical analyses were conducted using the R software (R Core Team 2021) and the packages ‘stats’, ‘lmerTest’, ‘emmeans’, ‘multcomp’, and ‘ggplot2’ (Hothorn et al. 2008; Wickham et al. 2016; Kuznetsova et al. 2017; Lenth 2021).

The statistical model used was as follows:

$$\:{Y}_{ijkl}=\:\mu\:+{s}_{m}{+t}_{i}+\:{r}_{j}+\:{c}_{k}+\:{e}_{ijkl}$$

Where: Yijklm = Observation of square m of ith treatment in row j column k from repetition l; µ = baseline mean; $\:{s}_{m}$= effect of latin square m; $\:{t}_{i}$ = effect of treatment I; $\:{r}_{j}$ = effect of period j; $\:{c}_{k}$ = effect of animal k; $\:{e}_{ijkl}$ = experimental error for ijkl.

## Results

### Intake and apparent digestibility

The level of TD inclusion in the diet did not have any significant effects on the sheep’s intake of DM, OM, CP, or NDF (*p* = 0.822) (Table 3). However, a linear effect was observed (R²=0.19; *p* = 0.006) in ADF intake as the TD inclusion in the diet increased. Compared to the animals on the TD0 diet, the ADF intake was 31.5% higher in the diet with the highest TD inclusion (*p* = 0.007).

The apparent digestibility of the diet decreased with the increasing inclusion of TD. Except from CP, a linear decreasing effect was observed on the DM (R²=0.42), OM (R²=0.47), NDF (R²=0.51), and ADF (R²=0.27) apparent digestibility coefficients (Table 3). When compared against TD0, the TD9 inclusion did not significantly affect nutrient apparent digestibility, however at TD27, the digestibility of DM, OM, NDF and ADF reduced by 7, 7, 21 and 24% respectively. Similarly, at TD45 the digestibility coefficients were 11, 12, 23 and 22% lower than in TD0, for DM, OM, NDF and ADF, respectively (Table 3).

**Table 3:** Intake and digestibility of sheep fed with increasing levels of *Tithonia diversifolia*

	TD0	TD9	TD27	TD45	SEM	*p*-value	L	Q	*R*²
Intake									
Dry matter (g d^− 1^)	1668	1744	1677	1805	116.6	0.822	0.49	0.75	
Dry matter (%LW)	2.42	2.59	2.56	2.65	0.225	0.908	0.63	0.88	
Organic matter (g d^− 1^)	1567	1607	1544	1638	105.4	0.922	0.73	0.88	
Crude protein (g d^− 1^)	322.2	307.6	300.9	332.3	23.24	0.774	0.72	0.55	
Neutral detergent fiber (g d^− 1^)	857.5	845.2	765.6	877.8	57.17	0.539	0.99	0.37	
Acid detergent fiber (g d^− 1^)	602.7	633.4	627.5	792.7*	24.59	0.028	0.007	0.17	0.19
Apparent digestibility coefficients									
Dry matter	0.68	0.69	0.64*	0.61*	0.011	< 0.001	< 0.001	0.65	0.42
Organic matter	0.71	0.71	0.66*	0.63*	0.011	< 0.001	< 0.001	0.74	0.47
Crude protein	0.74	0.73	0.70	0.71	0.014	0.260	0.279	0.44	
Neutral detergent fiber	0.62	0.60	0.49*	0.48*	0.018	< 0.001	< 0.001	0.17	0.51
Acid detergent fiber	0.50	0.52	0.38*	0.39*	0.026	0.002	< 0.001	0.38	0.27

*Within the same line indicates a significant difference from TD0; SEM = Standard error of the mean; p-value = Type I error rate of ANOVA; L, Q = p-value for linear and quadratic regression, respectively; R²=determination coefficient, showed only for significant regressions

### Nitrogen balance

The TD inclusion had no effects on the N balance of the evaluated sheep. No changes were observed in N intake, N excreted, or N retained among the diets (*p* > 0.05). In average, 46% of the N intake was retained, 28% was excreted in feces and 25% in the urine without significant differences among treatments (Table 4).

**Table 4:** Nitrogen balance of sheep fed with increasing levels of *Tithonia diversifolia*

	TD0	TD9	TD27	TD45	SEM	*p*-value	L	Q
N intake (g d^− 1^)	51.5	49.2	48.1	53.1	3.71	0.774	0.731	0.958
N excreted (g d^− 1^)	26.4	25.8	27.0	26.8	1.66	0.954	0.735	0.717
N faeces (g d^− 1^)	13.3	13.6	14.4	15.3	1.51	0.806	0.323	0.977
N urine (g d^− 1^)	13.0	12.2	12.6	11.6	0.86	0.730	0.553	0.652
N retained (g d^− 1^)	25.1	23.3	21.0	26.3	2.93	0.622	0.847	0.781
N retained / N intake (%)	47.5	47.2	43.0	48.1	3.03	0.621	0.912	0.566
N feces / N intake (%)	26.1	27.1	30.0	28.8	1.90	0.487	0.279	0.702
N urine / N intake (%)	26.3	25.6	26.9	23.1	2.70	0.759	0.576	0.708

*Within the same line indicates a significant difference from TD0; SEM = Standard error of the mean; p-value = Type I error rate of ANOVA; L, Q = p-value for linear and quadratic regression, respectively

No significant differences were observed in the NIDN content between the offered diets (Table 5) when expressed as a percentage of DM. However, the NIDN increased with the TD inclusion when expressed as a proportion of the total N of the diet (*P* = 0.045), although with a very low determination coefficient. On the other hand, the increasing level of TD in the diet did show a linear effect on the ADIN content when expressed both as proportion of DM and total N (*P* < 0.05). In both cases, the ADIN content in the TD45 diet was significantly higher than the diet without TD.

**Table 5:** Neutral and acid detergent insoluble nitrogen (NDIN, ADIN) content of diets with increasing levels of *Tithonia diversifolia*

	TD0	TD9	TD27	TD45	SEM	*p*-value	L	Q	*R* ^2^
NDIN (g kgDM^− 1^)	13.2	13.3	13.8	14.0	0.53	0.704	0.320	0.879	
NDIN (g kgN^− 1^)	473	452	486	524	17.9	0.056	0.045	0.357	0.09
ADIN (g kgDM^− 1^)	7.9	8.6	9.3	10.0*	0.46	0.029	0.008	0.771	0.18
ADIN (g kgN^− 1^)	286	290	328	373*	16.4	0.003	0.001	0.604	0.27

*Within the same line indicates a significant difference from TD0; SEM = Standard error of the mean; p-value = Type I error rate of ANOVA; L, Q = p-value for linear and quadratic regression, respectively; R²=determination coefficient, showed only for significant regressions

### Ruminal fermentation parameters

No effects were observed on the ruminal protozoa populations of the animals fed with TD (*p* > 0.05). The inclusion of TD in the diet, did not show any effects on the concentration of total SCFAs, propionate, butyrate nor A: P ratio from the animals (Table 6). However, a linear decrease was observed in the acetate molar proportions as the TD inclusion increased in the diet. When compared against the diet without TD the ruminal content of the animals fed with the TD45 inclusion had a 4.4% lower molar proportion of acetate (*p* = 0.008). Contrastingly, the molar proportions of all three iso-acids (valerate, iso-valerate and iso-butyrate) in the ruminal content of the animals increased linearly with the TD inclusion in the diet (*p* < 0.003); R^2^ = 0.28, 0.22 and 0.26, for the three iso-acids, respectively). When compared against the control, the ruminal content of the animals fed with TD45 inclusion had a 26.2, 29.1 and 79.8% higher molar proportions of valerate, iso-valerate and iso-butyrate, respectively (*p* < 0.05).

There was a negative linear relationship in the ruminal N-NH_3_ concentration due to the increase of TD in the diet (*p* = 0.011, R²=0.17). Animals fed with the highest level of TD (TD45) had a lower concentration (*p* < 0.05) of ruminal N-NH_3_ (18.9 mg dL^− 1^) compared to animals fed the TD-free diet (23.8 mg dL^− 1^).

**Table 6:** Ruminal fermentation parameters of sheep fed with increasing levels of *Tithonia diversifolia*

	TD0	TD9	TD27	TD45	SE	*p*-value	L	Q	*R*²
Protozoa (× 10^5^ mL^− 1^)	4.29	4.65	4.68	5.07	0.598	0.8371	0.612	0.996	
N-NH_3_ (mg dL^− 1^)	23.8	24.3	21.3	18.9*	1.29	0.0333	0.011	0.659	0.17
Total SCFA (umol mL^− 1^)	142.9	142.2	145.8	139.1	4.37	0.7562	0.780	0.603	
Acetate (mol 100 mol^− 1^)	65.7	65.7	64.0	62.8*	0.61	0.0083	0.003	0.811	0.22
Propionate (mol 100 mol^− 1^)	16.6	16.6	16.8	16.5	0.53	0.9778	0.993	0.703	
Butyrate (mol 100 mol^− 1^)	13.1	12.6	13.6	14.2	0.43	0.0888	0.099	0.651	
A: P ratio	3.99	4.00	3.84	3.81	0.142	0.6977	0.306	0.916	
Valerate (mol 100 mol^− 1^)	1.03	1.04	1.17	1.30*	0.048	0.0029	0.001	0.669	0.28
Iso-valerate (mol 100 mol^− 1^)	2.65	2.71	3.00	3.42*	0.198	0.0486	0.003	0.589	0.22
Iso-butyrate (mol 100 mol^− 1^)	0.94	1.24	1.34	1.69*	0.132	0.0078	0.001	0.937	0.26

*Within the same line indicates a significant difference from TD0; A: P = acetate to propionate ratio; SEM = Standard error of the mean; p-value = Type I error rate of ANOVA; L, Q = p-value for linear and quadratic regression, respectively; R²=determination coefficient, showed only for significant regressions. A: P = Acetate to propionate ratio

### Greenhouse gas emissions

No differences were observed in the emission rates of any of the evaluated gases among animals fed with different levels of TD (Table 7). Methane emissions for animals fed with TD ranged around 9.3 gCH_4_ kgDMI ^− 1^. Carbon dioxide emissions were close to 101 gCO_2_ kgDMI ^− 1^. Regarding N_2_O and NH_3_ emissions per kilogram of excreted N (feces + urine), animals fed with TD emitted an average of 2.62 gN_2_O and 37.4 gNH_3_. Considering the global warming potential for CO_2_, CH_4_, and N_2_O (Krey et al. 2014), the average emissions for animals fed with ruderal TD were around 153 kg of CO_2_ equivalents per animal per year.

**Table 7:** Mean emission rates of CH4, CO2, N2O and NH3 from sheep fed with increasing levels of *Tithonia diversifolia*

	TD0	TD9	TD27	TD45	SE	*p*-value	L	Q	
Methane									
g CH_4_ d^− 1^	12.41	12.55	13.17	12.25	0.449	0.5086	0.995	0.592	
g CH_4_ kgLW^− 1^	0.51	0.53	0.56	0.51	0.033	0.330	0.937	0.537	
g CH_4_ kgDMI^− 1^	8.52	8.76	10.28	9.05	0.958	0.421	0.460	0.250	
Carbon Dioxide									
g CO_2_ d^− 1^	128.59	126.21	138.14	140.10	7.00	0.4141	0.365	0.967	
g CO_2_ kgLW^− 1^	5.40	5.39	5.92	5.90	0.284	0.3824	0.449	0.846	
g CO_2_ kgDMI^− 1^	87.86	89.83	109.22	105.30	7.737	0.1906	0.141	0.515	
Nitrous Oxide									
mg N_2_O d^− 1^	64.87	65.08	70.12	63.50	2.034	0.1442	0.995	0.294	
mg N_2_O kgLW^− 1^	2.77	2.78	3.08	2.65	0.126	0.1290	0.929	0.378	
mg N_2_O kgDMI^− 1^	4.44	4.81	5.70	4.93	3.625	0.1293	0.456	0.252	
g N_2_O kg excreted N^− 1^	2.55	2.62	2.58	2.47	0.1284	0.8491	0.706	0.722	
Ammonia									
g NH_3_ d^− 1^	1.00	0.98	1.05	0.91	0.095	0.7985	0.790	0.699	
mg NH_3_ kgLW^− 1^	44.3	42.8	48.5	38.1	4.65	0.4790	0.733	0.565	
g NH_3_ kgDMI^− 1^	0.67	0.75	0.90	0.68	0.081	0.2052	0.876	0.305	
g NH_3_ kg excreted N^− 1^	39.0	39.1	37.3	35.8	4.04	0.9304	0.693	0.962	
Total emissions (kg CO_2_e year^− 1^)^a^	149.4	149.6	159.3	152.2	13.77	0.478			

^a^Calculated using IPCC (2019b) equation 10.21 A and 100-year global warming potential for CO_2_ (1), CH_4_ (21), e N_2_O (310) (Krey et al. 2014); d⁻¹ = per day; kgLW⁻¹ = per kilogram of live weight; kgDMI⁻¹ = per kilogram of dry matter intake; CO₂e = CO₂ equivalents

## Discussion

### Intake and digestibility

Normally, diets with higher content of ADF disappear more slowly from the rumen, which increases the retention time of the feed in it (lower passage rate), effectively reducing the DM intake of the animal as the feeling of physical satiety is prolonged (Silva 2006; Nikkhah 2014). In the present experiment, although animals on diets with TD45 showed higher ADF intake due to the higher proportion in the offered material, no significant differences were observed in DM or OM intake compared to animals fed with less TD. It is possible that despite having a higher ADF content, the particle size of the offered feed (~ 1 cm) was not a hindrance to the retention of the material in the rumen. Particle size and density are known determinants of passage rate (Clauss et al. 2011; Dufreneix et al. 2019). Valadares-Filho and Pina (2006) described that gas production from bacteria attached to food particles keeps them floating in the ruminal fluid, but once fermentation decreases due to particle degradation, their specific density increases, causing the material to settle in the ventral parts of the rumen, where it is susceptible to ruminal movements and escapes from the rumen. Thus, it is possible that the material with small particle size, fractionated during chewing, less fermentable due to the high ADF content and therefore with higher specific density, would precipitate in the rumen near the reticulo-omasal orifice and escape from the rumen more easily, increasing its quantity in the feces. This would be consistent with the lower apparent digestibilities observed in all the nutrients, except for CP.

Similar to the findings of the present experiment, Mahecha et al. (2008), Ribeiro et al. (2016) and Pazla et al. (2021) did not report differences in DM intake of cows fed increasing levels of TD, and Odedire and Olodi (2014) did not report differences in DM intake resulting from the inclusion of TD in goat diets. However, Fajemisin et al. (2013) and Castañeda-Serrano et al. (2018) reported increases in DM intake in diets with TD compared to control diets without it. The NDF content in the diet is a factor that determines the influence of the forage on dry matter intake. The inclusion of TD in the diets used by Fajemisin et al. (2013) and Castañeda-Serrano et al. (2018) significantly decreased the fiber content of the diet compared to the control without TD (559 vs. 707 g/kg of NDF (Castañeda-Serrano et al. 2018), and 12.96 vs. 25.98% of crude fiber (Fajemisin et al. 2013). In contrast, no changes in the NDF content were reported in the diets of Mahecha et al. (2008), Pazla et al. (2021), and Ribeiro et al. (2016) when compared against their respective controls.

### Protein digestibility and nitrogen balance

Despite the inclusion of TD in the diet, CP digestibility remained unchanged, consistent with the lack of significant difference in CP intake and CP apparent digestibility across the treatments. Protein was the only nutrient that did not show changes in its apparent digestibility with increasing TD inclusion and ADF intake. This outcome contrasts with other studies, which reported reduced fecal N excretion with increasing TD in diets and attributed this to increased intestinal digestibility of amino acids and greater ruminal CP solubility of diets with TD inclusion (Ramírez-Rivera et al. 2010; Yousuf et al. 2014; Castañeda-Serrano et al. 2018; Chacón-Góngora 2018; Durango et al. 2021). However, it is crucial to note that these studies utilized TD with different qualities from ours. Specifically, the NDF and ADF contents in these studies ranged from 350 to 440 g kg^− 1^ DM and 290 to 407 g kg^− 1^ DM, respectively. In contrast, our study’s ruderal wild growing TD had considerably higher NDF and ADF contents (670 and 560 g kg^− 1^ DM, respectively), likely due to the advanced vegetative stage of the material used (post-flowering). The high fiber content of our TD suggests a lower rumen degradability, which theoretically should result in higher CP output in the feces and a reduced digestibility coefficient. Surprisingly, this was not observed in our results; the N quantity in the feces remained unchanged with TD inclusion, indicating that CP digestibility was not impaired.

One plausible explanation for this is the potential protection of CP from rumen fermentation. This protection might be due to the high passage rate through the rumen (due to the small particle size) and to the protein being linked to indigestible fiber, as evidenced by the increase in ADIN as the TD inclusion increased. It is known that feed protein can escape rumen fermentation by both being associated to the cell structure and by reducing the feed retention time in the rumen (Church 1979; Sniffen et al. 1992; Owens et al. 2014).

We hypothesize that, partially degraded feed escapes from the rumen into the abomasum due to a rapid passage rate, the partially degraded fiber, permits the mostly undegraded protein to become exposed to digestive acids, allowing for enzymatic hydrolysis and subsequent duodenal absorption (Sniffen et al. 1992; Redfearn and Jenkins 2015). Consequently, the amount of protein in the feces remains low, maintaining overall protein digestibility, as observed. Different from protein, this rapid passage rate would have no noticeable effects on fiber digestibility as practically no fiber gets degraded in the abomasum (Church 1979), thus maintaining a high proportion in feces and consequently resulting in the observed low fiber digestibility.

The reduced levels of ruminal N-NH_3_ could potentially support this hypothesis, suggesting an overall decrease in ruminal protein degradation with the increase in TD inclusion. However, the increase in branched-chained volatile fatty acids (BCVFA) could suggest that at least some of the protein was degraded in the rumen, but also it could be a consequence of a reduction in fibrolytic microorganisms. These microorganisms, which typically use BCVFA as growth factors or carbon skeletons (An et al. 2024), could have been reduced due to insufficient ruminal N-NH_3_, this reduction in fibrolytic microorganisms would leave more BCVFA unconsumed, leading to the observed increase. Regardless of the specific mechanisms behind the BCVFA increase, it is likely that TD’s presence in the diet could have been responsible for it, as TD is reported to contain high amounts of branched-chained aminoacids (BCAA) (Fasuyi and Ibitayo 2011; Oluwasola and Dairo 2016), known precursors of BCVFA in the rumen (Slyter et al. 1979; Andries et al. 1987; An et al. 2024).

Overall, these findings suggest that the protein in TD is not less digestible, despite its high fiber content. Instead, the protein seems to escape ruminal degradation and becomes available for enzymatic digestion in the abomasum, thus supporting the N balance without altering protein digestibility.

### Greenhouse gas emissions

It is not possible to state that GHG emissions from sheep are dependent on the level of TD included in the diet since no significant relationship was found between the inclusion level and the gases measured in this study. It was hypothesized that the indirect effect of TD on ruminal mechanisms providing hydrogen as a substrate for enteric CH_4_ production was one of the ways by which the plant could mitigate CH_4_ production by means of reducing protozoa populations. But there was no evidence for this in the present experiment. It has also been mentioned that the tannin content in TD could have a detrimental effect on ruminal protozoa populations (Galindo et al. 2011, 2012, 2016; Delgado et al. 2012). Since the TD use in the present experiment had a low tannin content, and that the tannin content of the diet was not significantly different between treatments, this could be consistent with the lack of effects on the protozoa populations and therefore on the CH_4_ emissions, assuming that this was the only method in which the TD could have impacted methane production in the rumen.

Carbohydrate fermentation into acetate is a known route for hydrogen production, while fermentation into propionate is considered a ruminal sink for hydrogen (Getachew et al. 1998; Danielsson et al. 2017). The similarity of SCFA concentrations between diets, particularly in the A: P ratio, is consistent with the absence of significant differences in CH_4_ and CO_2_ emissions found in the present study. Methane production is often positively associated with the A: P ratio (Greening et al. 2019). The observed results coincide with those found by Ribeiro et al. (2016), who, using up to 150 g kg^− 1^ TD inclusion in the diet of dairy cows, also did not find differences in CH_4_ emissions, but emphasized that the A: P ratio between treatments was lower with TD inclusion. The average CH_4_ emissions found here for animals on the TD-free diet (12.59 gCH4 day^− 1^) are similar to those reported by (Lima et al. 2018, 2020) for Santa Inês sheep (14.14 gCH4 day^− 1^) and lower than the standard emission factors for sheep reported by IPCC methodologies (IPCC 2019a) of 5 to 9 kg of CH_4_ per head per year, compared to the 4.60 kg CH_4_ per head per year observed in the present study. It is important to note that the IPCC itself mentions that there can be high uncertainty in these values (50% variation).

It has been mentioned that excreta deposition increases N_2_O emissions due to intensified processes of nitrogen mineralization, nitrification, and denitrification (Cai et al. 2017), and that the decomposition of urea and undigested proteins present in animal feces and urine are potential sources of NH_3_ (Behera et al. 2013). Thus, it is expected that the lack of differences in N_2_O and NH_3_ emissions between treatments is consistent with the similarity in N excreted by animals on different diets (Table 3). The observed N_2_O emissions fall within the ranges reported by the IPCC (IPCC 2019b) for sheep (0.4–3.9 gN_2_O per kg of excreted N). According to the IPCC (IPCC 2019b), on average, 0.197 kg of NH_3_ are volatilized per kg of N deposited in excreta. The results found in this study are lower (0.039 kg of NH_3_), but the uncertainty reported by this organization includes a range from 0 to 0.295 kg of NH_3_ per kg of N applied to the soil.

The findings suggest that the inclusion of ruderal TD in sheep diets does not significantly affect GHG emissions, including CH_4_, N_2_O, and NH_3_. The lack of changes in CH_4_ emissions aligns with the stable acetate-to-propionate ratio and the low tannin content of the TD used. Additionally, the similarity in N excretion between treatments corresponds with the comparable N_2_O and NH_3_ emissions observed. These results indicate that while ruderal TD inclusion influences certain ruminal fermentation parameters, it does not lead to a noticeable reduction in overall GHG emissions from sheep.

## Conclusion

The inclusion of ruderal *T. diversifolia* in sheep diets demonstrated that while it does not significantly alter DM or OM intake, its high fiber content may limit nutrient absorption, particularly in terms of fiber digestibility. Interestingly, CP digestibility remained stable across all inclusion levels, suggesting that the protein in TD may escape ruminal degradation. Despite these effects on digestibility, ruderal TD as a forage source does not effectively mitigate GHG but neither does contribute to exacerbating CO_2_, CH_4_, N_2_O, or NH_3_ emissions. Under our conditions, replacing up to 27% of the Tifton hay with ruderal TD is recommended as it strikes a balance between nutrient intake and digestibility, maintaining effective protein utilization while avoiding the more pronounced reductions in fiber digestibility seen at higher inclusion levels. Future research should corroborate the observed stability in protein digestibility by investigating how *Tithonia diversifolia* protein behaves in the rumen, its degradation, binding to fiber, and potential bypass. Studies using higher-quality TD (e.g., pre-flowering harvest) should also assess whether it influences N_2_O emissions differently, to better define its role as a low-input mitigation option.

## Data Availability

The data that support the findings of this study are available from the corresponding author, SPM, upon reasonable request.
